# Evaluation of Brain Functions in Conversion Disorder with PET/MRI

**DOI:** 10.1192/j.eurpsy.2022.573

**Published:** 2022-09-01

**Authors:** S.Z. Tatlı, E. Özkan, M. Araz, M.İ. Erden, V. Şentürk Cankorur

**Affiliations:** 1 Ankara University Faculty of Medicine, Psychiatry, Ankara, Turkey; 2 Ankara University Faculty of Medicine, Nuclear Medicine, Ankara, Turkey; 3 Ankara University Faculty of Medicine, Radiology, Ankara, Turkey

**Keywords:** Neuroimaging, Conversion Disorder, Brain metabolism, PET/MRI

## Abstract

**Introduction:**

Since there is no objective criteria, unique clinical symptom or laboratory test to make the diagnosis of conversion disorder; its diagnosis and treatment is challenging which leads to a poor prognosis.

**Objectives:**

The aim of this study is to investigate the brain metabolic activity of patients with conversion disorder with PET/MRI.

**Methods:**

12 conversion disorder patients were included. Somatosensory Amplification Scale, Somatoform Dissociation Scale, Patient Health Questionnaire-15, Toronto Alexithymia Scale were filled in by the participants. Neurological, mental status examinations, Wechsler Adult Intelligence Scale-Revised Form (WAIS-R) and brain F18-FDG-PET/MRI were performed. Structured Clinical Interview for DSM-5, Hamilton Depression and Anxiety Scales were administered.

**Results:**

83% of the patients were female, the mean age was 33 years and average education period was 10,2 years. WAIS-R total scores were consistent with low avarage intelligence level.Cerebral hypermetabolism was detected in the primary visual cortex. Average regional brain metabolic activity had a tendency to increase in bilateral prefrontal, right sensorimotor (SM),cingulate,right inferior parietal,occipital lateral,right temporal lateral cortices and cerebellum. Each region was metabolically correlated with the homologous contralateral regions. Significant correlations in the same direction was found between frontal and occipital lateral & primary visual cortices; cerebellum and left sensorimotor cortex; anterior cingulate cortex(ACC) and superior parietal cortex & cerebellum. No correlations were found between ACC and left SM cortex.

**Conclusions:**

Findings of our study indicate that there are moderate changes in regional brain metabolic activities and inter-regional correlations in patients with conversion disorder. In order to confirm these findings, furter functional neuroimaging studies are needed.

**Disclosure:**

No significant relationships.

